# The Structural Basis of African Swine Fever Virus pS273R Protease Binding to E64 through Molecular Dynamics Simulations

**DOI:** 10.3390/molecules28031435

**Published:** 2023-02-02

**Authors:** Gen Lu, Kang Ou, Yiwen Jing, Huan Zhang, Shouhua Feng, Zuofeng Yang, Guoshun Shen, Jinling Liu, Changde Wu, Shu Wei

**Affiliations:** 1 Key Laboratory of Livestock Infectious Diseases, Ministry of Education, Shenyang Agricultural University, No. 120, Dongling Road, Shenhe District, Shenyang 110866, China; 2 The Preventive and Control Center of Animal Disease of Liaoning Province, Liaoning Agricultural Development Service Center, No. 95, Renhe Road, Shenbei District, Shenyang 110164, China

**Keywords:** pS273R protease, molecular docking, E64, molecular dynamic simulation, African swine fever virus

## Abstract

Identification of novel drugs for anti-African swine fever (ASF) applications is of utmost urgency, as it negatively affects pig farming and no effective vaccine or treatment is currently available. African swine fever virus (ASFV) encoded pS273R is a cysteine protease that plays an important role in virus replication. E64, acting as an inhibitor of cysteine protease, has been established as exerting an inhibitory effect on pS273R. In order to obtain a better understanding of the interaction between E64 and pS273R, common docking, restriction docking, and covalent docking were employed to analyze the optimal bonding position between pS273R−E64 and its bonding strength. Additionally, three sets of 100 ns molecular dynamics simulations were conducted to examine the conformational dynamics of pS273R and the dynamic interaction of pS273R−E64, based on a variety of analytical methods including root mean square deviation (RMSD), root mean square fluctuation (RMSF), free energy of ligand (FEL), principal component analysis (PCA), and molecular mechanics/Poisson–Boltzmann surface area (MM/PBSA) analysis. The results show that E64 and pS273R exhibited close binding degrees at the activity center of ASFV pS273R protease. The data of these simulations indicate that binding of E64 to pS273R results in a reduction in flexibility, particularly in the ARM region, and a change in the conformational space of pS273R. Additionally, the ability of E64 to interact with polar amino acids such as ASN158, SER192, and GLN229, as well as charged amino acids such as LYS167 and HIS168, seems to be an important factor in its inhibitory effect. Finally, Octet biostratigraphy confirmed the binding of E64 and pS273R with a KD value of 903 uM. Overall, these findings could potentially be utilized in the development of novel inhibitors of pS273R to address the challenges posed by ASFV.

## 1. Introduction

African Swine Fever (ASF) is a highly fatal, contagious, infectious disease that affects both domestic pig and wild boar [1]. This illness is caused by African Swine Fever Virus (ASFV), which first emerged in Kenya, East Africa, in 1921 and later spread to South Africa, the Caribbean, South America and Russia [2]. Since 2018, ASF outbreak has been ongoing in China, which affects more than 50% of the world’s pig population [3]. Up until June 2021, over 170 cases of ASF were officially recorded in China [4,5]. The virus is becoming a global threat. However, no effective vaccine or antiviral drug has been developed to combat the spread of ASFV [6,7]. Therefore, there is an urgent need to develop novel therapeutic strategies against this virus.

ASFV is the sole member of the genus *Asfivirus* within the family *Asfarviridae* [8]. The ASFV genome size ranges from 170 to 194 kb and encodes at least 150 polypeptides (30–50 of which are components of the virus particle) [9]. They have an important role for virus survival and transmission in its hosts. In particular, two polyproteins, (i.e., pp62 and pp220), which are expressed late during infection, can be cleaved to generate six structural components of the virus particle [10]. Of these, pp220-derived p14, p34, p37 and p150, and pp62-derived p15 and p35 are the main components of the core shell. This proteolytic process is crucial for assembling mature virus particles [11].

The ASFV pS273R protease can cleave the polyproteins pp62 and pp220 into different mature proteins [12], which is crucial for the organization of the core shell of ASFV [13]. It consists of two distinct domains (i.e., “core domain” and “arm domain”) [14], and its active site is located on the “core domain” with a catalytic triad of C232-H168-N187. Therefore, the protein cleavage of pp62 and pp220 can be blocked by effectively inhibiting the active site of ASFV pS273R proteinase. This will ultimately lead to the assembly of virus particles that lack the nucleocapsid, formation of defective icosahedral virions, and neutralization of the abnormally defective icosahedral virus particles [11,14]. The protein pS273R of ASFV has two functional regions. The first is a unique arm domain of unknown function, while the second is a core domain that contains all active sites for the hydrolysis of pp62 and pp220. The catalytic triplet Cys232-His168-Asn187, which is located in the core region, is responsible for peptidase hydrolysis activity of pS273R. Up to the current time, only a small number of molecules have been reported to inhibit pS273R, including the commercial small molecule inhibitor E64 [15], the defensin-like peptide toxin OPTX-1 [16], and a group of peptidomimetic aldehyde compounds designed based on the co-crystal of pS273R (6LJB, 6LJB) with SUMO-1 and its substrate (1TGZ: Structure of Human Senp2 and SUMO-1 Complex) [14,17].

Commercialized E64 inhibitor has shown potential as pS273R inhibitor. E64 is a cysteine inhibitor derived from *Aspergillus japonicus* that has been shown to inhibit a variety of cysteine proteases [18,19]. It has the inhibition feature of cysteinase activity to non-mammalian species. However, it does not affect the activity of cathepsin cysteinase in mammals [20]. Combining the crystal structure of the complex of E64 molecule and cysteine enzyme and previous studies on E64 molecule and its analogues [21,22,23,24,25,26], we hypothesized that C2 epoxide group of E64 reacts with the thiol group of cysteine through electrophilic attack, leading to the formation of a covalent bond with the catalytic triad of cysteine. This bond irreversibly inactivates the hydrolytic activity of the cysteine protease. As such, investigating the mechanism and dynamic response of E64 to pS273R can provide insight into the impact of E64 inhibitors on the conformation and enzymatic activity of pS273R protease, and ultimately facilitate the optimization and design of more effective and safer pS273R inhibitors to address the significant challenges posed by ASFV.

## 2. Results

### 2.1. Model Evaluation of ASFV pS273R Modeling Structure

**Figure 1:**
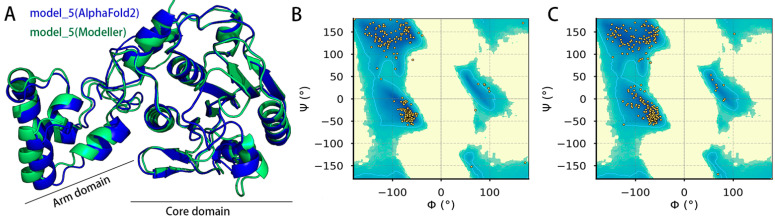
Modeling structure of pS273R. (**A**) Structural model of pS273R produced by ColabFold-alphaFold2 (blue, model_5) and Modeller 10.4 (green, model_5). (**B**) Ramachandran plot of pS273R model by Modeller 10.4. (**C**) Ramachandran plot of pS273R model by ColabFold-alphaFold2.

The pS273R model generated by Modeller 10.4 and designated as model_5, has the lowest discrete-optimized-protein-energy (DOPE) score. Meanwhile, the pS273R model generated by AlphaFold2 and designated as model_5, has the highest confidence level. The root mean square deviation (RMSD) of alpha carbon atoms for both models is 0.829 Å, as shown in Figure 1A. The model of pS273R generated by AlphaFold2 exhibits a high score combining with the data of the Ramachandran plot, SAVES v6.0, and DeepUMQA v3.0. The Ramachandran plot shows that model_5 generated by AlphaFold2 has a high percentage (94.8%) in the favorable interval. The model scores from ERRAT, VERIFY 3D-1D and Global lDDT are 98.49%, 97.80%, and 85.87%, respectively (Figure 1B,C, Table 1).

### 2.2. Molecular Docking Analysis of the ASFV pS273R and E64

**Figure 2:**
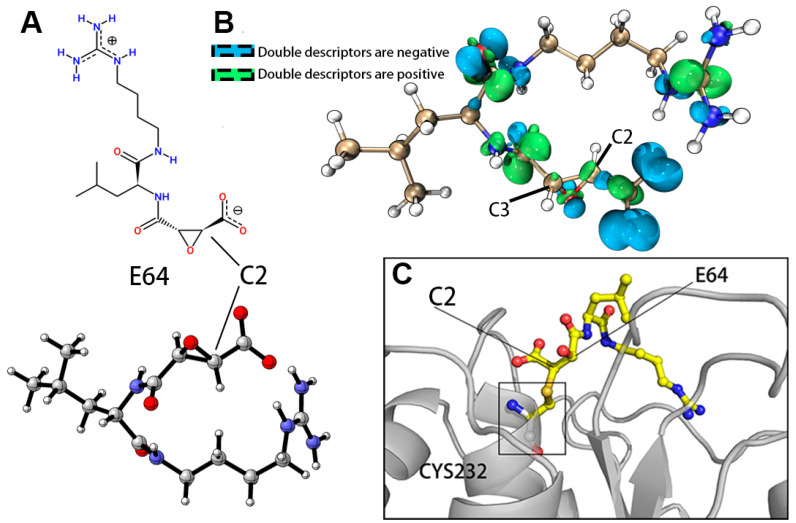
The molecular and optimized structures of the E64 inhibitor. (**A**) 2D and 3D structures of E64 optimized by quantum chemistry program (ORCA 5.03, Theoretical level BLYP-D3/def2 SVP def2/J CPCM (water)). (**B**) Isosurface Plots of the dual descriptors (orbital weights and Fukui functions) for the E64 molecule. The positive and negative isosurface values are directly set as 0.02 and −0.02 respectively. (**C**)The C-S covalent bond between CYS232 and E64. CYS-232 is displayed with white carbon skeleton and sulfur atoms are shown in yellow.

The dual descriptors of E64@C2 (C2) and E64@C3 (C3) are both positive and showing certain electrophilicity and local Lewis acid characteristics, while C2 shows a larger and more pronounced positive region, which indicates a stronger electrophilic attack capability of C2, although the difference is not as significant (Figure 2). visual analysis is somewhat ambiguous and subjective. Therefore, we assigned dual descriptors (Fukui functions and orbital weights), Hirshfeld charges, and synergistic local electrophilic/nucleophilic indices to C2 and C3 of E64 to show the extent to which the C2 and C3 atoms act as catalytic reaction sites (Table 2). The Hirshfeld charges, f- (condensed Fukui functions) and nucleophilic index of C2 atom were all higher than those of C3, which was consistent with the observation of the orbital weight double descriptor that the C2 atom had a higher priority in electrophilic attack than C3. The C2 atom of E64 will be treated as the covalently bonded atom in subsequent analysis.

The first five poses of E64 and pS273R common docking have a docking score below −7 Kcal/mol. When the distance between E64 @ C2 and CYS232 @ S was limited to 3.5 Å, the first five posts remained a lower Docking score (<−6 Kcal/mol), with a docking score of −3.8 Kcal/mol for the covalent combination (Table 3). The molecular and optimized structures of the E64 inhibitor are shown in Figure 2A.

**Figure 3:**
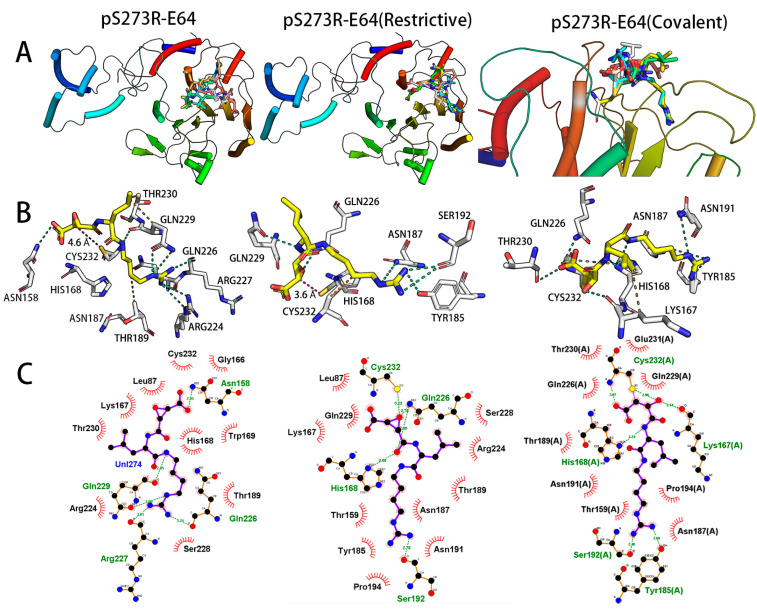
Interaction diagrams of molecular common docking, restricted docking, and covalent docking of pS273R and E64. (**A**) Docking pose of E64 molecules with top five docking scores in pS273R. (**B**) The interaction of E64 molecules with amino acids in the active pocket of pS273R mapped using the open-source version of Pymol 2.6. Hydrogen bonds are shown in green and hydrophobic interactions in yellow. The carbon skeleton of the E64 molecule colored yellow, the amino acid carbon skeleton is colored white, the nitrogen atoms are colored blue, and the oxygen atoms are colored red. (**C**) The 2D interaction of E64 molecule with amino acids in the active pocket of pS273R was plotted using the EDU version of LigPlot+ v.2.2. The carbon backbone of the inhibitor molecule is shown in purple, nitrogen atoms in blue, oxygen atoms in red, residues involved in hydrogen bond interactions named in green, and residues in red named as those that provide pockets and hydrophobic interactions.

Additionally, the molecular docking results showed that E64 showed good interaction with pS273R in all three docking modes, as shown in Figure 3. The first five poses bound to pS273R protein and occupied the pS273R active pocket in both non-covalent and covalent docking (Figure 3A). In the common docking mode, E64 formed five hydrogen bonds with ASN158, GlN226, Arg227 and GlN229 of pS273R, and C2 is just 4.6 Å away from CYS232 @ S. The restricted docking case E64 forms five hydrogen bonds with CYS232, HIS168, SER192 and GlN226. The distance between C2 and CYS232 @ S is 3.5 Å E64 is bonded to CYS232, E64 forms six hydrogen bonds with HIS168, LYS167, SER192, TYR185, and CYS232 (Figure 3B). The 2D interaction pattern visualizes the interaction of E64 with pS273R in the three docking modes (Figure 3C).

### 2.3. Molecular Dynamics Simulation and Affinity Calculation of pS273R and E64 Complex

**Figure 4:**
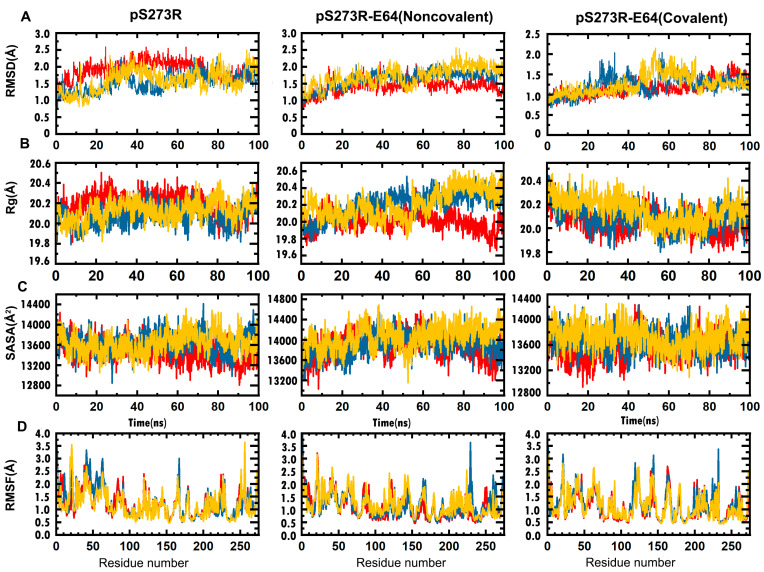
Molecular dynamics simulations of free pS273R, ASFV pS273R and E64 complexes for 100 ns duration. (**A**) The convergence of pS273R, pS273R−E64 noncovalent docking complex, and pS273R−E64 covalent docking complex in MD simulation process were verified by RMSD. (**B**) The structural compactness analysis of MD simulation by Rg curves between pS273R, pS273R−E64 noncovalent docking complex, and pS273R−E64 covalent docking complex. (**C**) The analysis of solvent-accessible surface area in the MD simulation between pS273R, pS273R−E64 noncovalent docking complex, and pS273R−E64 covalent docking complex. (**D**) Behavior and stability of each residue in the MD simulation process between pS273R, pS273R−E64 noncovalent docking complex, and pS273R−E64 covalent docking complex through RMSF. Among them, Trajectory 1, Trajectory 2 and Trajectory 3 are colored red, yellow, and blue, respectively.

The molecular dynamics simulation of pS273R in the free state and its covalent and non-covalent system complexes with E64 was conducted with three parallel groups and the simulation duration was 100 ns. The initial velocities for each group were varied in order to study the stability and dynamic behavior of the systems in a solvent environment. All of the systems reached equilibrium after 70 to 80 ns. The root mean squared deviation (RMSD) of the pS273R showed large oscillations (ranging from 1 to 2.5 Å) at the beginning of the simulation, then the RMSD for Trajectory 2 and Trajectory 3 decreased and converged to 0.16 nm after 70 ns. The RMSD of the between pS273R−E64 non-covalent complex remained stable after 60 ns, with the RMSD for Trajectory 1, Trajectory 2, and Trajectory 3 converging to 1.47, 1.76, and 1.86 Å, respectively. The pS273R and E64 covalent complex reached equilibrium fastest for trajectory 1, while the RMSD values for trajectory 2 and 3 had a wide range of 45 to 75 ns and 25 to 70 ns, respectively. After 75 ns, the RMSD values for all three samples stabilized at around 1.49, 1.32, and 1.40 Å, respectively. The radius of gyration (Rg) values for the three samples in each simulation group generally followed similar trends. Trajectory 1 of the pS273R−E64 non-covalent complex showed a much lower Rg value than Trajectory 2 and Trajectory 3 after 60 ns. At the same time, the Rg values for Trajectory 2 and Trajectory 3 were larger than all samples of the pS273R and the pS273R−E64 covalently complex. The solvent-accessible surface area (SASA) of pS273R was fairly consistent with that of the pS273R−E64 covalently complex, with a small fluctuation around 13,600 Å^2^. When E64 was bound to pS273R in a non-covalent manner, the SASA values for all samples were larger than those of the pS273R and the pS273R−E64 covalently complex. The root mean squared fluctuation (RMSF) of the residues of pS273R in the free state varied among the different samples, but the ARM region displayed high flexibility, with LYS21, GLU41, and GLN63 all showing high RMSF values greater than 3 Å. The pS273R−E64 non-covalent complex increased the flexibility of the GLN229 and VAL120-LYS144 regions, and the covalent binding of E64 to pS273R further enhanced this trend, leading to a significant increase in the flexibility of the VAL120-LYS167 region and a decrease in the flexibility of the ASP245-ALA273 region (Figure 4A–D).

**Figure 5:**
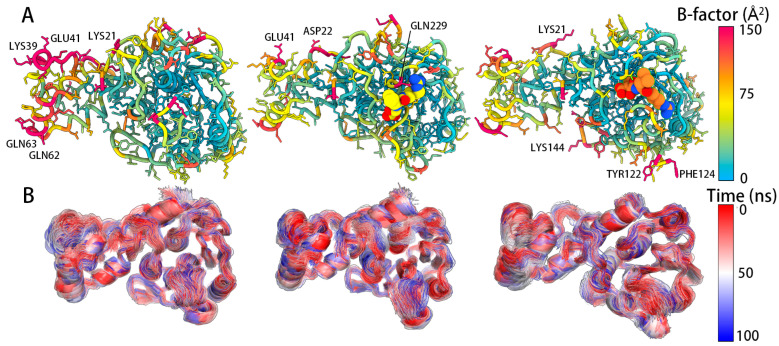
B-factor (Transformed by RMSF) and motion trajectories of pS273R−E64 complexes and pS273R. (**A**) B-factor mapped using chimera X for pS273R of free state and covalent and noncovalent pS273R−E64 complexes. (**B**) Trajectories for pS273R of free state and covalent and noncovalent pS273R−E64 complexes (merged trajectories).

Subsequently, the RMSF is transformed into B factor by gmx_rmsf instruction after the trajectories were merged. The ARM domain of pS273R in the free state showed high flexibility. However, E64 was bound to pS273R, the flexibility of the ARM domain was significantly reduced. The binding of E64 and pS273R forms steric hindrance to amino acids near the active pocket, reducing the flexibility of the surrounding amino acids. Additionally, the RMSD, Rg, SASA, and intermolecular hydrogen bonds of each group of simulations were calculated, as shown in Table 4. The results clarified that E64 and pS273R formed stable hydrogen bonds. An average of up to 4.6 and 6.0 hydrogen bonds between E64 and pS273R were recorded in the noncovalent and covalently bound states (Figure 5).

### 2.4. The Analysis of pS273R and pS273R−E64 Complexes in Different Bonding States Based on Gibbs Free Energy Landscape and Principal Component Analysis

**Figure 6:**
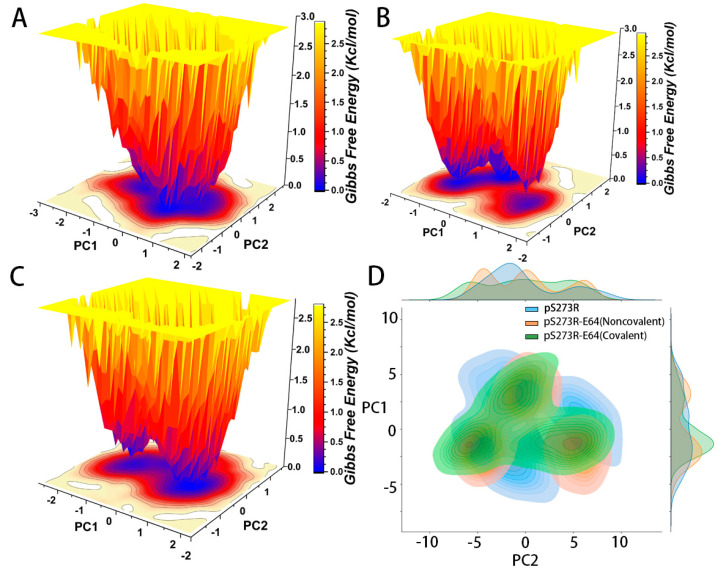
The analysis of pS273R and pS273R−E64 complexes in different bonding states based on FEL (Kcal/mol) and PCA. (**A**) FEL of pS273R in the free state. (**B**) FEL of pS273R−E64 (Noncovalent). (**C**) FEL of pS273R−E64 (Covalent). (**D**) The analysis for pS273R and pS273R−E64 complex based on PCA.

Protein motion information extracted from the merged trajectories was projected onto the principal components (PC1 and PC2), and the Gibbs free energy of the system was approximated by gmx sham. The Gibbs free energy landscape for the free state of pS273R showed three energy depressions and was compartmentalized by a <0.45 Kcal/mol potential. Meanwhile, pS273R−E64 complex (non-covalent) showed one major energy depression and two indistinguishable low potential energy regions two low potential energy regions. For pS273R−E64 (covalent), it showed two energy depressions and a low potential energy region that was difficult to distinguish, and the two main energy basins were distributed along PCA2. The results of FEL and external PCA were consistent. The free state pS273R has the widest conformational space, and the pS273R−E64 complex in the binding state has a very similar PCA, it affected the Gibbs free energy landscape of pS273R in different degrees, which means that, when E64 was combined with pS273R, the conformational space of pS273R was changed (Figure 6).

### 2.5. Gibbs Binding Free Energy Calculation and Decomposition of pS273R−E64 Complex Based on Molecular Mechanics Poisson–Boltzmann Surface Area

**Figure 7:**
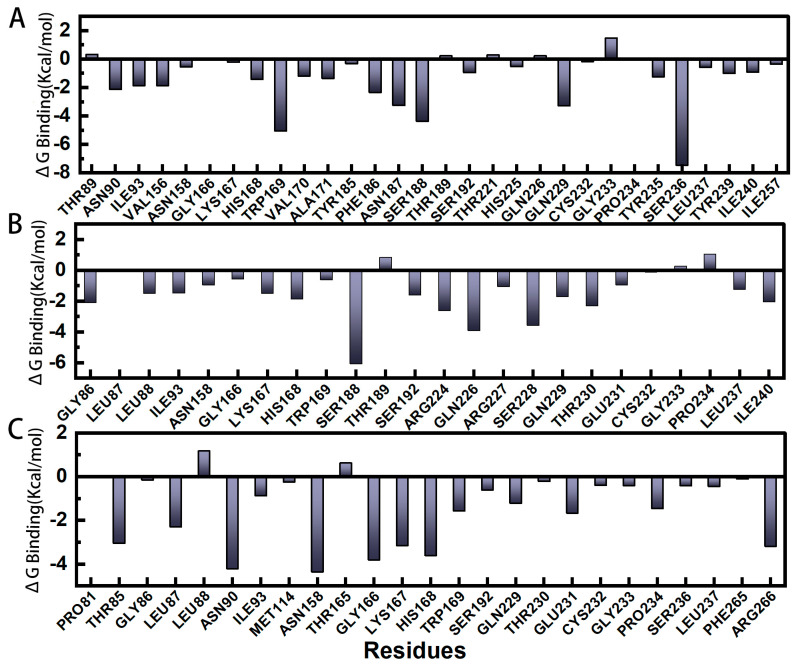
The Gibbs binding free energy decomposition diagram of pS273R−E64 complex based on (MM−PBSA). (**A**–**C**) are trajectory 1, trajectory 2 and trajectory 3, respectively.

Molecular mechanics/Poisson–Boltzmann surface area (MM/PBSA) is a valid and reliable method for calculating the binding free energy of small molecule inhibitors and their target proteins. As shown in Table 5, the calculation of Gibbs binding free energy showed that the binding of E64 inhibitor was mainly from van der Waals forces, meanwhile, electrostatic and hydrophobic effects were also important components of Gibbs binding free energy. pS273R−E64 (non-covalent complex) had ΔG Bindings of the three trajectories were −6.67 Kcal/mol, −8.76 Kcal/mol, and −11.62 Kcal/mol, respectively. Additionally, the three parallel groups of ΔE vdw, Δ Eelec and ΔG nonpol were negative, indicating that ΔE vdw, ΔE elec and ΔG nonpol were favorable for the non-covalent binding of E64 and pS273R, while the entropy contributed unfavorably to ΔG Binding. Trajectory 1 had the highest -TΔS of 7.16 kcal/mol. Further decomposition of the Gibbs binding free energy will be benefit for understanding the contribution of each residue to ΔG Binding. The contribution of residues to ΔG Binding is inconsistent from sample to sample. Residues of PRO 81-ILE 93 located in the loop-region at the entrance of the active pocket provide part of the attraction. However, ASN158, LYS167, HIS168, TRP169, SER192 and GLN229 all produced negative Gibbs binding free energy contributions in the three simulated systems with different initial velocities, and these residues formed stable interactions with E64 (Figure 7).

### 2.6. Effect of E64 on ASFV pS273R Proteinase Affinity

**Figure 8:**
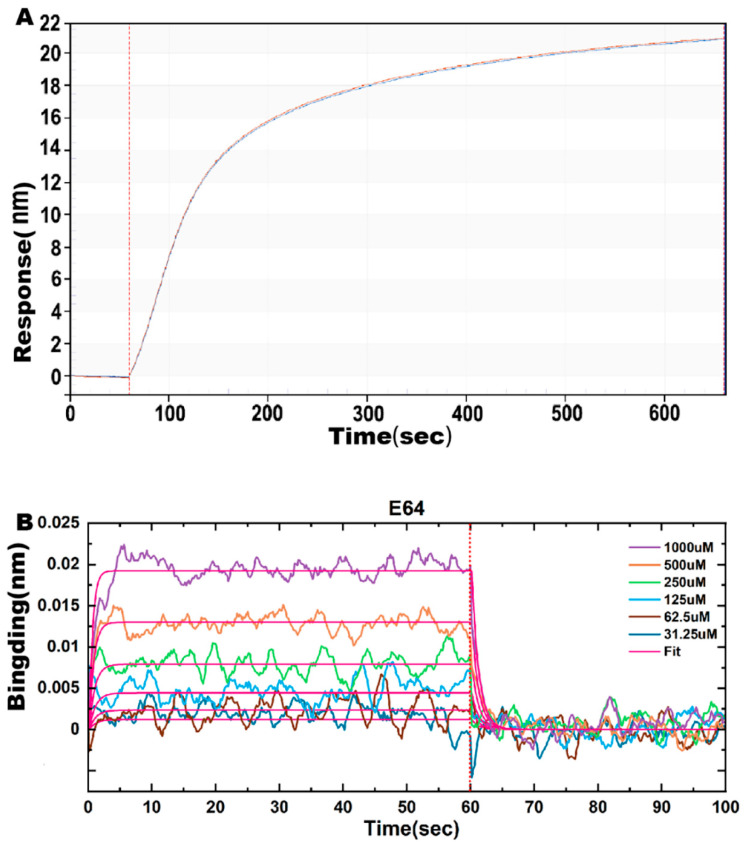
The affinity effect of ASFV pS273R and E64. (**A**) The biotin−ASFV S273R protein was solidified with SSA sensor with a solidifying signal of about 20 nm. (**B**) The SSA sensor immobilized with the ASFV S273R protein was subjected to binding dissociation detection with different concentrations of E64 molecules at baseline 60 s, association 60 s, and dissociation 60 s. The kinetic curves were obtained and analyzed with Octet Analysis 12.0 software to obtain the kinetic data.

The result of biotin-ASFV S273 protein solidified with SSA sensor formed a well-fitted curve, which displayed that biotin-ASFV S273R protein and SSA sensor had a good curing effect (Figure 8A). In addition, the KD value of the SSA- biotin-S273R protein and small molecule E64 was 903 uM based on the Formula (KD = kdis(1/s)/kon(1/Ms), indicating that ASFV S273R protein could bind to E64, and both had good affinity (Figure 8B, Table 6).

## 3. Discussion

To evaluate the binding pattern of pS273R and E64, we calculated the dual descriptors (Fukui function and orbital weight). Hirshfeld charge and local electrophilic/nucleophilic index of the E64 molecule using computational chemistry. The C2 atom was more active than the C3 atom. This is also consistent with the trend of reactive energy barriers in electrophilic attacks of E64 and its analogue E64C [23,24]. Then, according to the model data predicted by AlphaFold2, three methods of molecular common docking, restricted docking and covalent docking were used to analyze the binding properties. Additionally, the dynamic behavior of covalent and non-covalent pS273R−E64 complexes in solvent were studied by molecular dynamics simulations, which allowed us to gain a better understanding of the interactions between E64 and pS273R, as well as the behavior of the complexes in solution. The restricted docking method can be used to approximate the near attack state of the covalent bonding reaction between E64 and pS273R at a low cost [27]. The Gibbs binding free energy of pS273R−E64 can be quantified using the MM/PBSA method and decomposed into residues to reveal the relative importance of amino acids involved in E64 binding. Moreover, in view of its proteinase functioning and catalytic property, in vivo affinity assay of ASFV pS273R and E64 by Octet^®^ Biolayer Interferometry was performed to evaluate the degree of binding between E64 and ASFV pS273R.

The docking results indicate that E64 has a strong interaction with pS273R under the three docking modes. In the unrestricted docking mode, the distance between E64@C2 and the sulfhydryl group of CYS is only 4.6 angstroms, indicating a strong interaction. Even when the distance of the reactive group is restricted to 3.5 angstroms, E64 and pS273R still interact well. Although this distance is not sufficient for a nucleophile reaction, the binding conformation of E64 and pS273R at this distance is similar to that of the reaction under real conditions. The hydrogen bonding of GLN226, HIS268 and SER192 to E64 helped to stabilize the conformation of E64, and the covalent docking results showed that E64 still had a strong interaction with pS273R after the formation of covalent bonding [28].

Molecular dynamics simulations show that all three simulated systems reach equilibrium after 80 ns, despite inconsistent initial simulation speeds. In addition, the RMSD of the free state pS273R has a large oscillation amplitude in the early stage, indicating that the free state pS273R has a wider conformation. Notably, the ARM domain of pS273R showed high flexibility in the free state, but the binding of E64 reduced its flexibility, which is consistent with the observed results of PCA and FEL. Although the binding mode of the inhibitor was different from that of the pS273R substrate. Preliminary speculation is that the activity of the ARM region of pS273R is related to the binding of the pS273R substrate. The ARM region is distant from the binding site of pS273R. However, its remote regulation may have a crucial impact on the activity of the protein. The flexibility of some residues in the core domain of pS273R is altered after E64 binding to pS273R. LYS167 in the loop region (THR159-LYS167) reduces the flexibility of THR165. Though covalently bound E64 reduces the RMSF in the loop region, it increases the flexibility of Glu142-Lys144. Based on the eutectic structure of the homologous enzyme (PDB: 1EUV) and substrate in the RCSB PDB database [29], it is inferred that the activity characteristics of Glu142-Lys144 in the ring region may be related to the non-covalent binding of the pS273R shear substrate, which may affect the binding of pS273R to the substrate. The covalent bonds formed between E64 and Cys232 occupy the active pocket of ASFV pS273.

Gibbs binding free energy calculations indicate that the binding of the E64 inhibitor primarily results from van der Waals forces, while electrostatic and hydrophobic interactions also contribute to the Gibbs binding free energy. The van der Waals energy (E vdW) has a greater contribution to the energy compared to ΔE elec, which may be due to the presence of the hydrophobic group n-butyl in the E64 molecule’s structure and the hydrophobic backbone of the E64 molecule. ΔE elec may be associated with the formation of a large number of hydrogen bonds between E64 and pS273R key residues. The entropic contribution is highly detrimental to the stability of the system, even in agreement with the net binding free phase energy of the system. In this system, the entropic contribution is approximated by the Interaction Entropy method [30], which is considered to be reliable in terms of computational efficiency and numerical value. A larger fluctuation in the interaction energy implies a greater entropic loss in the binding free energy, which implies a larger fluctuation in the binding energy of E64 with pS273R. E64 is a flexible molecule, though the structure of the E64 molecule has been optimized under aqueous solvent based on thee simulated system in advance, E64 binds in the open pocket of pS273R, and the solvent effect may have a significant impact on the E64 binding to pS273R. Optimization of the structure of E64 to increase the rigidity of E64 molecules, such as shortening the flexible backbone, is likely to increase the inhibitory activity of E64 molecules against pS273R. In addition, E64 analogs are also likely to have inhibitory activity against pS273R [31,32], which is worth exploring. A group of uncommercialized covalent inhibitors of aldehydes that were reported to accompany the crystal structure of pS273R also have significant inhibitory effects on pS273R, and the CHO warheads of such inhibitors were retrofitted to the substrate analogs [14]. However, they also face challenges of cytotoxicity, hepatic and renal metabolic toxicity in clinical trials and commercialization, and peptide inhibitors may be at risk of degradation by other intracellular protease systems that do not consistently inhibit the activity of pS273R. At the same time, covalent inhibitors are a double-edged sword: off-target toxicity may bring disastrous consequences [33]. Perhaps the synthesis of non-covalent D-type amino acid substrate analog inhibitors would be a good choice [34,35].

The energy breakdown of MM/PBSA and each residue reveal the relative importance of the different components of the binding energy and the amino acids involved in binding. Gibbs free energy decomposition showed different residue contributions among the three samples, but ASN 158, LYS167, HIS168, TRP 169, SER192 and GLN229 all produced negative Gibbs binding free energy contributions in the three simulation systems with different initial velocities, indicating that these residues form stable interactions with E64. This is important for the persistence of the catalytic ability of E64 to bind and inhibit pS273R, and a molecule/potential drug with similar ability may bind and inhibit pS273R (even without necessarily forming a covalent bond with CYS). It was noted that the soft tick-derived OPTX-1 and the Ixodes derived defensin-like peptide also produced reversible and non-covalent competitive inhibition of pS273R [35]. Molecular docking showed that OPTX-1 might form hydrogen bonds with SER192, ARG224, GLN229, THR164 and ASP165 around the active pocket of pS273R, occupying the active pocket of pS273R. Interestingly, SER192 and GLN229 were consistent with the results of our simulation analysis, further suggesting that SER192 and GLN229 are key amino acids for pS273R targeted inhibition. In addition, the simulations also show that pS273R has a very flexible ARM region. Its function is still unknown. It is interesting to note that the degree of flexibility of the ARM region is influenced by whether the core domain binds molecules. The ARM domain of pS273R is unique, and there is a flexible region on the pocket of pS273R of the loop region. With more detailed PCA analysis and conformational clustering methods, it is feasible to identify conformational changing switches and design allosteric inhibitors [36,37].

As an in vitro real-time biomolecular interaction instrument, Octet^®^BLI can detect the interaction between molecules quickly and sensitively and calculate the binding and dissociation constants accurately. Furthermore, Octet^®^ BLI systems enable real-time, label-free analysis for the determination of kinetics and affinity, also providing robust monitoring of biomolecular interactions to overcome limitations in throughput, performance and cost associated with traditional technologies such as Western blot. These fluidics-free, low-maintenance, sensitive systems increase lab productivity, reduce costs and shorten experimental timelines [38]. Moreover, as revealed by Octet^®^ Biolayer Interferometry studies, it was observed that a good affinity exists between ASFV pS273R and E64 at baseline 60 s, association 60 s, and dissociation 60 s. Although the measured Kd values still differ from the Gibbs free energy calculated by MM/PBSA, this is not alarming, and in most cases the MM/PBSA method can only approximate the Gibbs binding energy [39], and the combination of pS273R and E64 is further validated.

Taken together, these findings will help us understand the effects of E64 on the hydrolytic activity of pS273R and the conformation dynamics of pS273R protein and provide new insights into the structure and function of cysteine proteases, which will guide researchers to optimize and design safer and more efficient inhibitors of pS273R. Nevertheless, an inevitable limitation of this study is that we did not conduct the in vitro and in vivo experiments of live ASFV. In the near future, we will conduct subsequent validation studies with the Harbin Veterinary Research Institute, which can provide a foundation for the treatment and control of ASF.

## 4. Materials and Methods

### 4.1. Homology Modeling and Structural Evaluation

Homology modeling was performed according to the crystal structure of ASFV pS273R (PDB: 6LJB) from the ASFV Georgia 2007/1 strain using classical homology modeling and artificial intelligence-based methods. The pS273R protein sequence from the Georgia 2007/1 isolate (YP_009927235) was obtained from the NCBI database. Homology modeling was performed by MODELLER 10.9 [40], and five conformations were generated using the RCSB PDB:6LJ9 template. Subsequently, the models were ranked according to their DOPE scores, and the optimal model was selected for energy minimization in Chimera 1.16 [41]. The AlphaFold2 ColabFold online tool also generated a total of five conformations [42,43], and enabled the Use amber option to eliminate the possibility of unreasonable sidechain collisions. The model with the highest (pIDDT) score was chosen to further research. Both models were examined and evaluated by the Ramachandran plot, SAVES 6.0 (https://servicesn.mbi.ucla.edu/SAVES/ accessed on 16 November 2022) contains the VERIFY and ERRAT [44,45,46,47], and DeepUMQA 3.0 [48], and then, the optimal conformations were selected for further calculations.

### 4.2. Molecular Optimization and Molecular Docking of E64

The structure file of E64 molecule in SDF format was retrieved from PubChem, and the protonic state of E64 was corrected in Avogadro, and followed by the use of ORCA 5.0.3 with DFT-D3 correction, def2-SVP orbital basis set, def2/J auxiliary basis set and theoretical level [49,50,51]. The geometrical structure of E64 was optimized in aqueous solution (CPCM solvent model) [52]. After checking that there was no virtual frequency in the result file, the wave functions of N, N + 1 and N − 1 electronic states of the E64 molecule were calculated in ORCA (theoretical level: B3LYP/6-31G+) [49,53,54], and then the electrophilic reactivity of E64@C2 (C2) and E64@C3 (C3) atoms of the E64 molecule in Multiwfn 3.8 was analyzed [55,56], including Hirshfeld charge, condensed Mr Fukui function and condensed local electrophilic/nucleophilic index [57,58,59]. The pdbqt file of the protein receptor and candidate molecules was created using MGLTools-1.5.7 [60]. The charge and rotation bond number were recorded in the pdbqt file of the candidate molecules. The ULPS instruction was used in this process to ensure that the information of non-polar hydrogen was completely recorded in the pdbqt file. Use the getbox plugin in pymol to obtain the docking box coordinates (X = −9.2, Y= 4.3, Z =−8.6).

Molecular docking and restricted docking were performed with the watvina procedure, adding an implicit water model to approximate solvation. Restricted docking applied additional parameters to limit the distance between the S atoms of CYS232 and E64@C2 to 3.5 angstroms. Covalent docking was performed with ADFR 1.2 [61], the epoxide group of the E64 molecule was turned on, and the E64@C2 atom acted as the covalently bonded atom. The S atom of CYS232 of pS273R was reprobated, and AutoGridFR was used to create a trg file containing the details of the receptor, covalent residues, maps and other docking information.

In the calculation process of molecular docking, semi-flexible docking was adopted, that is, the target protein pS273R was always rigid, and all torsion bonds of the inhibitor could rotate freely. After the docking was completed, scores were made according to the affinity order, and the reasonable posture of interaction was judged according to experience. Finally, the optimal pS273R−E64 complex was selected to determine the conformation and spatial coordinates of the inhibitor as the initial conformation of MD simulation.

### 4.3. Molecular Dynamics Simulations

Molecular dynamics simulations and the insights into protein motion often play important roles in drug discovery. The GROMACS 2022.3 suite was applied for free pS273R, simulation of pS273R−E64 (non-covalent) and pS273R−E64 (covalent) complexes [62]. Restrained ElectroStatic Potential charges (RESP)were fitted by Multiwfn 3.8 (dev) basing on the result files generated by ORCA 5.0.3 (optimizing E64) [49,56], and the files of parameter and topology for the ligands were produced by Ambertools [63]. Amber14sb force field was used to describe all atomic interactions throughout the simulation [64]. The water model TIP3P was performed to simulate the dissolution of the system [65], and sodium and chloride ions were added to the simulation system with approximate the 0.15M NaCl sodium chloride concentration under the physiological state. The simulation system minimizes energy through two steps of steepest descent method and conjugate gradient method. Each simulation system is balanced by NVT of 300 ps (equal particle number N, volume V, temperature T) and NPT of 300 ps (equal particle number N, pressure P, temperature T). The Particle-Mesh-Ewald (PME) method was used for the long range electrostatic interaction in the system [66]. The linear constraint solver (LINCS) algorithm was applied to constrain the bond length of heavy atoms [67]. The Verlet truncation scheme was used for calculating the short-range interaction, the cutoff value (cutoff) of the short-range van der Waals interaction and the electrostatic interaction with14 A. In this process, the Parrinello–Danadio–Bussi temperature coupling algorithm [68] and the Parrinello–Rahman pressure coupling algorithm were employed [69]. After completing the necessary equilibria in the md stage, random initial velocities are generated for each system by using parameters (gen_vel = yes and gen_temp = 300). Three parallel simulations were carried out for each simulation system of pS273R and its covalent and non-covalent complexes with E64. (Parallel groups 1,2 and 3 of each simulation system were trajectory 1, trajectory 2 and trajectory 3, respectively.)

The simulation time step was set to 2 fs, and each simulation system was sampled three times, meanwhile, the built-in program of gromacs was employed to analyze RMSD, RMSF, Rg and FEL. After combining the tracks, the data were extracted through mdtraj and conducted the analysis of PCA based on sklearn 1.0. The results were visualized by Pymol 2.6 [70], chimera X 1.5 [71], origin pro 2021, matplotlib 3.6.2 and seaborn 0.12.1 [72].

The MM/PBSA method used to provide more details on the ligand receptor interaction [39]. This method is a reliable and efficient model widely used to calculate the binding free energy of non-covalent binding complexes [73]. Subsequently, gmx_MMPBSA was used to calculate the Gibbs free energy of E64 and pS273R for non-covalent interactions [74,75].

### 4.4. Affinity Assay of ASFV pS273R and E64

Octet^®^ Biolayer Interferometry (BLI) was used to detect the affinity of E64 and ASFV pS273R. After biotin treatment of ASFV S273R recombinant protein, PBS was selected as buffer solution to dilute it to 50 ug/mL, and biotin-S273R protein was solidified with Super Streptavidin (SSA) sensor for 10 min with a solidifying signal of about 20 nm. Next, E64 at 1000 um was diluted 2-fold with PBST + 5% DMSO, resulting in concentration gradients of 1000, 500, 250, 125, 62.5 and 31.25 um. The SSA sensor immobilized with the ASFVS 273R protein was subjected to binding dissociation detection with different concentrations of E-64 molecules at baseline 60 s, association 60 s, and dissociation 60 s. The kinetic curves were obtained and analyzed with Octet Analysis 12.0 software to obtain the kinetic data.

## 5. Conclusions

In summary, the findings indicate that combination of E64 and pS273R is stable through molecular dynamics simulation of covalent and noncovalent complexes of pS273R−E64. E64 has the abilities of specific binding to the catalytic triad and, in turn, inhibits the activity of ASFV pS273R protease. Hence, this research can be used as the structural basis for further optimizing inhibitors and developing anti ASFV therapeutic drugs. It is hoped that our findings can pave the way for future development of novel molecular inhibitor and effective treatment strategy to target this harmful pathogen.

## Figures and Tables

**Table 1:** Evaluation and scoring of the pS273R model.

Model Quality Assessment Method		Model_5 (Modeller)	Model_5 (AlphaFold2)
Ramachandran plot	Favored regions	96.8%	94.8%
	Allowed regions	3.2%	5.2%
SAVES v6.0	ERRAT	78.87	98.49
	VERIFY(3D-1D score)	89.74%	97.80%
DeepUMQA v3.0	Global lDDT	73.81	85.87
	Global lDDT (Refined)	82.21	82.43

**Table 2:** Activity descriptors of C2 and C3 atoms for E64 inhibitors calculated by density functional theory.

Atom	Hirshfeld Charges (e)			Condensed Fukui Functions (e)			Condensed Local Electrophilicity/Nucleophilicity Index (e*eV)	
	q (N)	q (N + 1)	q (N − 1)	f-	f+	f0	Electrophilicity	Nucleophilicity
C2	0.0233	0.0042	0.0512	0.0279	0.0191	0.0235	0.01034	0.10878
C3	0.0209	0.0035	0.0403	0.0194	0.0174	0.0184	0.00938	0.07557

Note: N, N + 1, and N − 1 represent the different electronic states of the E64 molecule (N, net charge: 0 spin multiplicity: 1. N + 1, net charge: -1 spin multiplicity 2. N − 1, net charge: 1 spin multiplicity: 2.). The ∗ in e∗ eV represents a multiplication sign.

**Table 3:** Docking scores for the top five poses of the common, restricted, and covalent docking results of pS273R with E64 molecules.

Pose Number	Docking Score (Kcal/mol)		
	Molecular Docking (Common)	Molecular Docking (Restrictive)	Molecular Docking (Covalent)
1	−7.85	−6.91	−3.8
2	−7.49	−6.62	−3.5
3	−7.47	−6.44	−3.0
4	−7.35	−6.35	−3.1
5	−7.08	−6.30	−2.4

**Table 4:** Molecular dynamics simulations of RMSD, Rg, SASA, RMSF and intermolecular hydrogen bonds extracted from three sets of pS273R, pS273R and E64 (non-covalent/covalent) complexes at 100 ns duration.

		RMSD (Å)	Rg (Å)	SASA (Å^2^)	H-bone
pS273R	Trajectory 1	19.1 ± 0.23	20.17 ± 0.011	13372.7 ± 183.7	-
	Trajectory 2	16.7 ± 0.21	20.09 ± 0.010	13648.2 ± 227.5	-
	Trajectory 3	16.7 ± 0.26	20.11 ± 0.010	13716.4 ± 206.3	-
pS273R−E64 (Noncovalent)	Trajectory 1	14.7 ± 0.16	20.00 ± 0.011	13874.2 ± 252.7	3.209 ± 1.142
	Trajectory 2	17.6 ± 0.12	20.29 ± 0.008	13970.8 ± 188.9	4.662 ± 1.012
	Trajectory 3	18.6 ± 0.25	20.30 ± 0.016	14122.4 ± 227.1	2.886 ± 1.284
pS273R−E64 (Covalent)	Trajectory 1	14.9 ± 0.18	20.02 ± 0.010	13598.5 ± 171.9	6.087 ± 1.468
	Trajectory 2	13.2 ± 0.19	20.05 ± 0.010	13614.6 ± 191.9	4.776 ± 1.240
	Trajectory 3	14.0 ± 0.23	20.08 ± 0.012	13655.6 ± 189.2	2.692 ± 1.036

**Table 5:** Gibbs binding free energy (kcal/mol) of pS273R−E64 calculated by MM/PBSA.

MM/PBSA (Kcal/mol)	Trajectory 1	Trajectory 2	Trajectory 3
ΔE vdw	−45.84 ± 3.17	−46.25 ± 3.02	−49.17 ± 2.81
ΔE elec	−17.66 ± 3.64	−35.54 ± 6.28	−25.40 ± 4.24
ΔG pol	22.93 ± 2.92	46.15 ± 5.73	38.32 ± 2.90
ΔG nonpol	−33.66 ± 0.77	−33.87 ± 1.35	−35.97 ± 0.91
ΔEDISPER	55.45 ± 0.88	56.55 ± 1.43	58.82 ± 0.92
ΔGGAS	−63.50 ± 2.34	−81.78 ± 6.97	−74.57 ± 2.49
ΔGSOLV	44.72 ± 2.96	68.82 ± 5.65	61.17 ± 3.32
-TΔS	7.16 ± 0.06	6.29 ± 0.06	4.64 ± 0.05
ΔG Binding	−11.62 ± 2.87	−6.67 ± 3.87	−8.76 ± 2.69

Note: ΔE vdW, van der Waals energy. ΔE elec, electrostatic energy. ΔG pol, polar solvent energy. ΔG nonpol, Non-polar solvent energy. ΔEDISPER, dispersion term. ΔGGAS, Total gas phase free energy. ΔGSOLV, Total solvation free energy -TΔS, Interaction Entropy. ΔG Binding, Total energy.

**Table 6:** The affinity of E64 for ASFV S273R.

Variant	KD (M)	Kon (1/Ms)	Kdis (1/s)
E64	9.027 × 10^−4^	1.014 × 10^3^	9.150 × 10^−1^

## Data Availability

The data that support the findings of this study are available from the corresponding author upon reasonable request.

## Sample Availability

Samples of the compounds and the data that support the findings of this study are available from the authors.
